# COVID-19 hotspots through clusters analysis in France (may–October 2020): where should we track the virus to mitigate the spread?

**DOI:** 10.1186/s12889-021-11857-8

**Published:** 2021-10-11

**Authors:** Guillaume Spaccaferri, Clémentine Calba, Pascal Vilain, Loïc Garras, Cécile Durand, Corinne Pilorget, Nahida Atiki, Pascale Bernillon, Laëtitia Bosc, Erica Fougère, Jean-Baptiste Hanon, Valérie Henry, Caroline Huchet-Kervella, Mélanie Martel, Valérie Pontiès, Damien Mouly, Enguerrand Rolland du Roscoat, Stéphane Le Vu, Jean-Claude Desenclos, Anne Laporte, Regional MONIC group, Patrick Rolland

**Affiliations:** Santé publique France, 12 Rue du Val d’Osne, 94410 Saint-Maurice, France

**Keywords:** COVID-19, Hotspots, Clusters, Descriptive analysis, Criticality, Public health

## Abstract

**Background:**

In France, the lifting of the lockdown implemented to control the COVID-19 first wave in 2020 was followed by a reinforced contact-tracing (CT) strategy for the early detection of cases and transmission chains. We developed a reporting system of clusters defined as at least three COVID-19 cases, within seven days and belonging to the same community or having participated in the same gathering, whether they know each other or not. The aim of this study was to describe the typology and criticality of clusters reported between the two lockdowns in France to guide future action prioritisation.

**Methods:**

In this study we describe the typology and criticality of COVID-19 clusters between the two lockdowns implemented in France (between May and end of October 2020). Clusters were registered in a national database named “MONIC” (MONItoring des Clusters), established in May 2020. This surveillance system identified the most affected communities in a timely manner. A level of criticality was defined for each cluster to take into consideration the risk of spreading within and outside the community of occurrence, and the health impact within the community. We compared the level of criticality according to the type of community in which the cluster occurred using Pearson’s chi-square tests.

**Results:**

A total of 7236 clusters were reported over the study period, particularly in occupational environment (25.1%, *n* = 1813), elderly care structures (21.9%, *n* = 1586), and educational establishments (15.9%, *n* = 1154). We show a shift over time of the most affected communities in terms of number of clusters. Clusters reported in occupational environment and the personal sphere had increased during summer while clusters reported in educational environment increased after the start of the school year. This trend mirrors change of transmission pattern overtime according to social contacts. Among all reported clusters, 43.1% had a high level of criticality with significant differences between communities (*p* < 0.0001). A majority of clusters had a high level of criticality in elderly care structures (82.2%), in disability care centres (56.6%), and health care facilities (51.7%).

**Conclusion:**

These results highlight the importance of targeting public health action based on timely sustained investigations, testing capacity and targeted awareness campaigns. The emergence of new SARS-CoV-2 variants strengthen these public health recommendations and the need for rapid and prioritise vaccination campaigns.

## Background

The coronavirus disease 2019 (COVID-19) is caused by severe acute respiratory syndrome corona virus 2 (SARS-CoV-2). The virus was first detected in Wuhan province, China, in December 2019. Since then, the epidemic has progressed rapidly into a pandemic. The disease can result in severe and even fatal respiratory diseases such as acute respiratory distress syndrome [1]. Thus, the pandemic led to an exponential growth in hospital admissions highlighting a risk of saturation of local intensive care units [2].

In France, the first wave of COVID-19 epidemic was controlled by a lockdown from March 17 to May 10, 2020, bringing all regions below the daily hospital admissions threshold of 1 per 100,000 inhabitants [2–4]. The lifting of the lockdown was followed by a resumption of activities and a reinforced contact-tracing (CT) strategy. This strategy aimed at the early detection of cases and transmission chains, including the follow-up of clusters. After a steady rise of the cases starting from August 2020, a second wave was observed leading to a second lockdown from October 30 to December 14 [5]. The second lockdown was lifted on December 15 and a national curfew was implemented.

The repetition of epidemic waves highlights the need for anticipating COVID-19 future hotspots in order to mitigate the spread and to limit the disease burden. The aim of the current study was to describe the typology and criticality of clusters reported between the first two lockdowns in France and their change over time to guide future action prioritisation.

## Methods

We defined a COVID-19 case as a person, symptomatic or not, with a positive SARS-CoV-2 RT-PCR on a nasopharyngeal swab. A cluster was defined as the identification of at least three COVID-19 cases, within seven days and belonging to the same community or having participated in the same gathering, whether they know each other or not. Santé publique France developed a guide for COVID-19 clusters’ investigation and evaluation, which is available online [6].

Clusters were detected through the CT strategy or voluntarily notified by affected communities. The CT strategy aimed at the identification of high-risk contacts (forward tracing) and did not target the origin of contamination (backward tracing). Clusters were registered at the regional level in a national database named “MONIC” (MONItoring des Clusters). All 18 French regions were involved in this surveillance, including the five overseas regions. Familial clusters (i.e. people sharing the same home) were not targeted in this surveillance because of the assumed low risk of spreading outside the family home. However, social gathering between several family branches were included. For each cluster, descriptive data were collected and regularly updated based on results of epidemiological investigations conducted by local health agencies, including: date of reporting, type and size of the exposed community, date of symptom onset (or date of sample) of the first and the latest cases, number of cases, hospitalisations and deaths.

The results of the investigations were used to define a level of criticality (low, moderate, high) for each cluster, according to qualitative and quantitative criteria (Table 1). These criteria allowed to take into consideration the risk of spreading within and outside the community of occurrence, and the health impact within the community. The risk of spreading was based on the size of the population that has been exposed (i.e. the larger the population, the higher the risk), and on difficulties to implement health management measures. This risk was used to refine the criticality level during investigations [6]. The level of criticality was regularly updated according to the evolution of the situation (e.g. number of reported cases). The criteria used to assess the level of criticality were included in MONIC and did not change during the study period.

**Table 1:** Epidemiological criteria to assess the level of criticality of the clusters

Epidemiological criteria	Category A	Category B	Category C
Number of cases	< 5	5 to 9	> 9
Ratio number for cases/community size	< 5%	10%	15%
Vulnerability factors	None	Medical^a^	Social^b^ and medical
Severity factors	No hospitalisation and no death	Less than 5 hospitalisations, no death	More than 5 hospitalisations and/or ≥ 1 death
Time period between last case onset of symptoms and signal	≤7 days	8 to 14 days	14 days
Risk of spreading	Low	Moderate	High
**Level of criticality**	**Low:** only category **A** criteria **Moderate:** at least one category **B** criteria without **C** **High:** at least one category **C** criteria		

^*a*^ *Population with elderly people, people with comorbidities or with immunodeficiency*

^*b*^ *Disadvantage and fragile environment, people with difficulty accessing health care, language and/or cultural barriers*

MONIC included data related to the community type where the cluster was reported (e.g. educational establishment, occupational environment) and the level of criticality. These data were analysed to describe the national distribution of reported clusters within communities, in total and over time. We compared the level of criticality according to the community type in which clusters occurred using Pearson’s chi-square tests (Stata V12).

## Results

### Distribution of clusters among communities over time

**Fig. 1:**
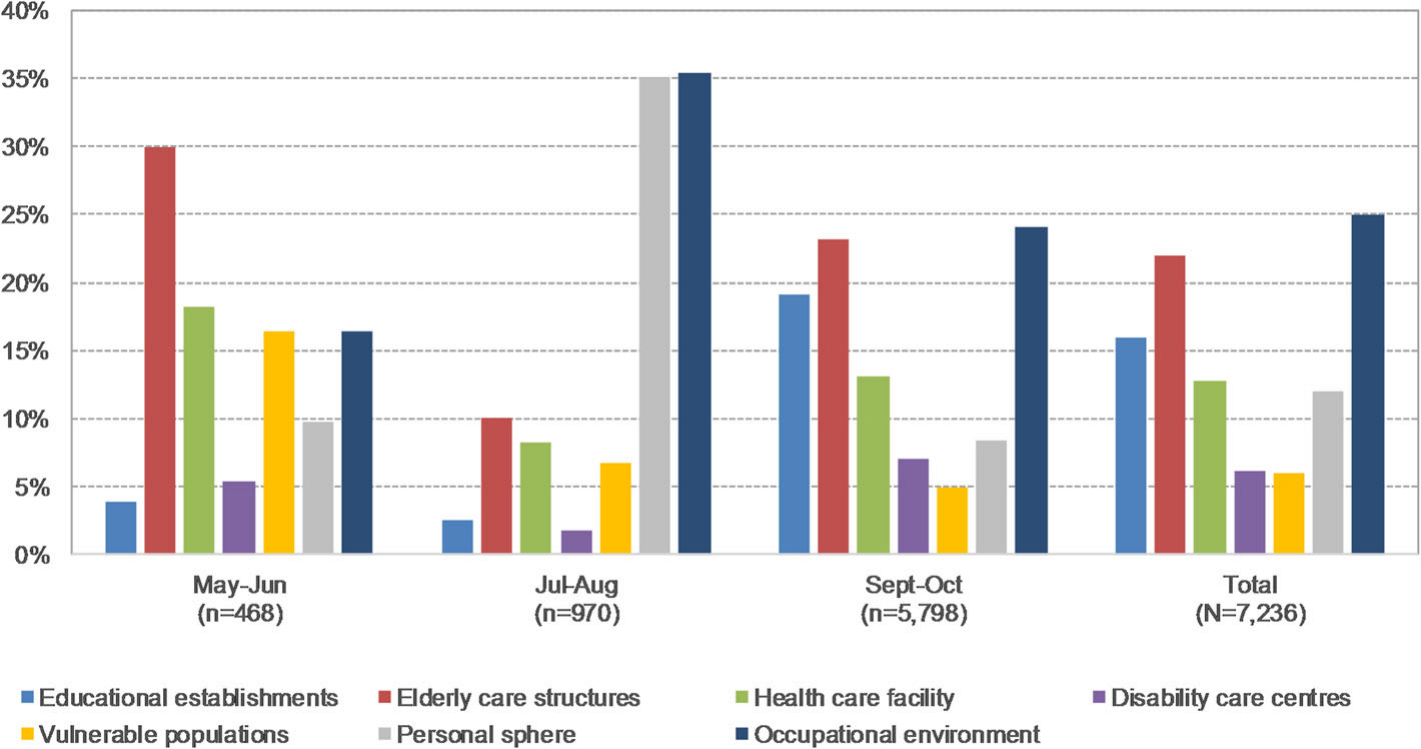
Distribution of reported clusters by community type, France, May–October 2020. Blue: Educational establishments; Red: Elderly care structures; Green: Health care facility; Purple: Disability care centres; Yellow: Vulnerable populations; Grey: Personal sphere; Dark blue: Occupational environment

Between May and October 2020, 7236 clusters were reported, particularly in occupational environment (25.1%, *n* = 1813), elderly care structures (21.9%, *n* = 1586), and educational establishments (15.9%, *n* = 1154) (Fig. 1).

The number of reported clusters and the distribution of affected communities changed over time. In May and June, following the lifting of the first lockdown, 468 clusters were reported. The proportion of clusters was the highest in elderly care structures (29.9%, *n* = 140), health care facilities (18.2%, *n* = 85), occupational environment (16.5%, *n* = 77), and vulnerable populations (migrants and travellers communities, penal institutions, social integration centres, and child social welfare structures) (16.5%, n = 77). During summer holidays (July–August), the number of clusters doubled (*n* = 970), mainly due to the abrupt onset of clusters in occupational environment (*n* = 343) and personal sphere (family meetings, social gatherings, and sporting activities) (*n* = 341), representing respectively 35.4 and 35.2%. Over the next period (September–October), the number of clusters increased at a faster pace (*n* = 5798) due to the emergence of outbreaks in educational establishments (19.2%, *n* = 1111), the resurgence of clusters in elderly care structures (23.2%, *n* = 1348) while contaminations in the occupational environment remained at a high level (24.0%, *n* = 1393).

### Relationship between level of criticality and community type

Among the 7236 reported clusters, 43.1% had a high level of criticality (from 19.6 to 82.2% according to the community type, *p* < 0.0001) and an average of 15 cases per cluster were reported (from 8 to 27) (Table 2).

**Table 2:**
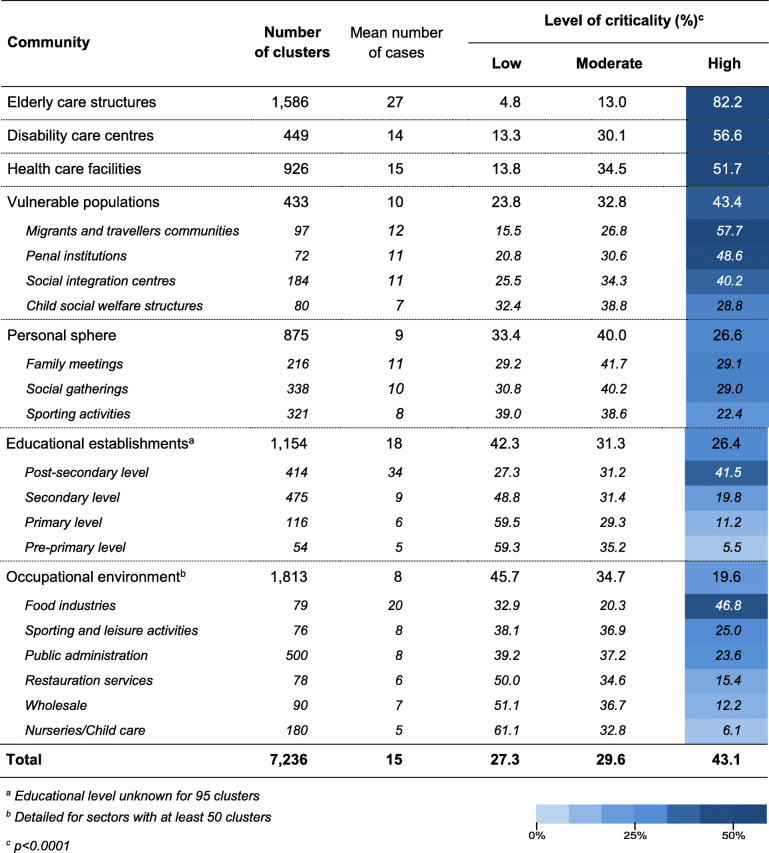
Communities with clusters reported, ranked by level of criticality, France, May–October 2020

A large majority of clusters (82.2%) had a high level of criticality in elderly care structures where an average of 27 cases were reported. To a lesser extent, most of clusters had a high level of criticality in disability care centres (56.6%) and health care facilities (51.7%) with an average of 14 and 15 cases respectively. Above 40% of clusters in vulnerable populations had a high level of criticality, particularly for migrants and travellers’ communities, penal institutions, social integration centres fostering people living in precarious situation, and homeless people. An average of 10 cases were reported in these communities, from 7 in child social welfare structures to 12 in migrants and travellers’ communities.

In educational establishments, the proportion of clusters at high criticality was much lower (26.4% with high criticality) but differed by educational level (*p* < 0.0001): it was less than 20% in secondary and lower level schools, whereas it was > 40% for post-secondary level which includes older people with greater social interaction. Disparities were also found in the average number of cases reported, from < 10 in secondary and lower level schools to 34 in post-secondary level.

Criticality of COVID-19 clusters varied by type of occupational environment (*p* < 0.0001): it was high for nearly 50% of clusters reported in food industries (46.8%) compared to ≤25% for the other sectors. Indeed, an average number of 20 cases were reported in food industries whereas it was < 10 in the other sectors. Although the total number of clusters reported in the public administration sector (e.g. various public services, local authorities, police, firefighters …) was large (*n* = 497), the level of criticality was mostly low to moderate with an average number of 8 cases per cluster. A large number of clusters (*n* = 875) was reported within the personal sphere but less than 30% of these had a high level of criticality, with an average number of 9 cases.

## Discussion

The number of clusters reported in France increased dramatically over time, in accordance with the epidemic dynamic, and the type of communities that were the most affected shifted over time. Elderly care structures, health care facilities, and vulnerable populations were among the most affected communities at the end of the first lockdown (May–June). These structures were also the most targeted by testing effort. The number of clusters increased thereafter (July–August) with the resumption of activities and social interactions, and the relaxation in the application of barrier measures [7]. The occupational environment and personal sphere may have thus facilitated the spread of the virus during this time [8]. The number of clusters amplified substantially in September, in conjunction with the end of summer holidays, the re-opening of schools and insufficiently protected (i.e. non-mandatory wearing of surgical mask), increased social interactions.

Clusters criticality was particularly elevated for communities with high prevalence of medical risk-factors but also for which health protection measures (physical distancing, wearing of masks) were difficult to implement (old age, disability, social deprivation …) [9]. These difficulties lead to a high number of cases reported both for patients/residents and social/health care workers (HCW). Heavy workload and understaffing faced by these workers may have also contributed to the dissemination of the virus within these communities.

High level of criticality reflects the impact within a community (i.e. severity) but also the risk of spreading to the general population. Among occupational environment, clusters that occurred in food industry plants and more specifically slaughterhouses very often had high level of criticality because of the high number of cases reported. This was mainly due to environmental factors and work conditions which favour the spread of the virus [10]. Similarly in post-secondary school where increased social interactions favoured multiple contaminations and hence the occurrence of clusters at high levels of criticality [11]. In the personal sphere, the level of criticality was lower but clusters were more numerous, particularly during summer.

Possible overlaps between clusters and communities may have occurred, especially in the personal sphere for which family meetings could also be wider social gatherings. The number of clusters in social gatherings was probably highly underestimated due to the difficulty of the CT program to identify chains of transmission among people that do not necessarily know each other, such as in bars, restaurants, fitness centres, public transports or cultural events. The number of cases reported for these clusters was probably also underestimated. Generally, the availability of COVID-19 biological tests may have had an impact on the number of cases reported in each cluster as well as the number of reported clusters by community type, irrespective of the region. Because of a lack of testing capacity in the initial stages, testing campaigns were mainly implemented in communities where CT was limited and/or for which the criticality could be greater. At a later stage, when the testing capacity had increased, testing campaigns were implemented at a larger scale.

Although our monitoring system had several limitations (including potential lack of completeness and representativeness), it has been helpful to target and adapt control efforts at the national and territorial levels. The analysis of data collected through this system helped in providing public health recommendations to better anticipate future actions. Furthermore, information related to contamination circumstances would increase the understanding of cluster occurrence.

The early detection of clusters and isolation of cases are of paramount importance to mitigate the spread [8, 12, 13]. Moreover, regular analysis of clusters should alert on specific situations (e.g. high number of clusters or cases in specific communities) to conduct targeted awareness campaigns. During high circulation, investigations of clusters should focus on hotspots to limit the epidemic impact. Testing campaigns targeting at-risk communities and populations with poor access to health care system should limit disease burden. These communities would also benefit from adapted support, human resources and appropriate equipment to better manage outbreaks.

The emergence of new SARS-CoV-2 variants strengthen these public health recommendations. Rapid and prioritised vaccination campaigns, targeting both patients/residents and professionals in hotspots remain a key point to mitigate spread and limit the burden of the virus.

## Conclusion

An increase of COVID-19 clusters was observed in France between the two lockdowns. There was a shift over time in the type of affected communities in relation to the population lifestyle dynamic. Hotspots were mostly found in communities with at-risk populations and/or where control measures were difficult to implement. Such communities should benefit from timely testing campaigns and granted of appropriate resources for rapid control including vaccination. When low virus circulation level is achieved, investigations of all clusters need to be sustained to mitigate the spread, with a special attention for most susceptible communities. Targeted prevention and control measures focusing on the most affected communities over time remain a key point to contain and flatten the COVID-19 curve. The advent of new SARS-CoV-2 variants and widespread availability of vaccines need to be taken into account to refine public health interventions aimed at controlling clusters of SARS-COV-2 intervention.

## Data Availability

The datasets generated and analysed during the current study are not publicly available due to the protection of health data regulation set by the French National Commission on Informatics and Liberty (Commission Nationale de l’Informatique et des Libertés, CNIL). Data can be available from the corresponding author after permission obtained from Santé publique France.

## Abbreviation

CT: contact-tracing; HCW: Health care workers
